# Geographic Disparities in County-Level Prevalence of Alzheimer’s Disease Across the United States

**DOI:** 10.1093/geroni/igaa057.3124

**Published:** 2020-12-16

**Authors:** Mackenzie Fowler, Michael Crowe, Richard Kennedy

**Affiliations:** University of Alabama at Birmingham, Birmingham, Alabama, United States

## Abstract

While national and state estimates of the prevalence and incidence of AD are available, estimates across finer geographic regions offer an opportunity to tailor programs to the needs of the local population. Previously, we estimated prevalence and incidence of AD at the county level across the continental United States and found that estimated prevalence of AD varied more than threefold across counties, predominantly in the Southeastern and Midwestern United States. We also observed “islands” of low AD within regions with high AD, and vice versa. We update these findings by examining changes in projected prevalence of AD over time, and comparing projected prevalence of AD to prevalence of AD diagnoses in Medicare. We also examine regional variation in provider specialty patterns and racial differences across counties as possible explanatory factors. Understanding small-area geographic disparities in prevalence will be critical for addressing practice variation in the prevention and diagnosis of dementia.

